# New endobronchial ultrasound (EBUS) techniques

**DOI:** 10.31744/einstein_journal/2022CE0159

**Published:** 2022-09-06

**Authors:** Guilherme Moratti Gilberto, Priscila Mina Falsarella, Eserval Rocha, Ricardo Mingarini Terra, Marcia Jacomelli, Rodrigo Gobbo Garcia

**Affiliations:** 1 Hospital Israelita Albert Einstein São Paulo SP Brazil Hospital Israelita Albert Einstein, São Paulo, SP, Brazil.

**Figure 1:**
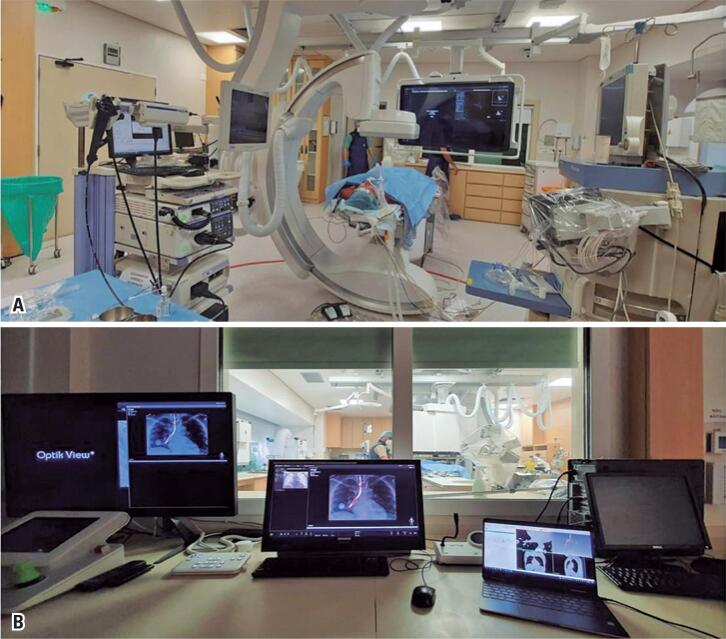
(A) Interventional radiology suite; (B) Control room of the interventional radiology suite

**Figure 2:**
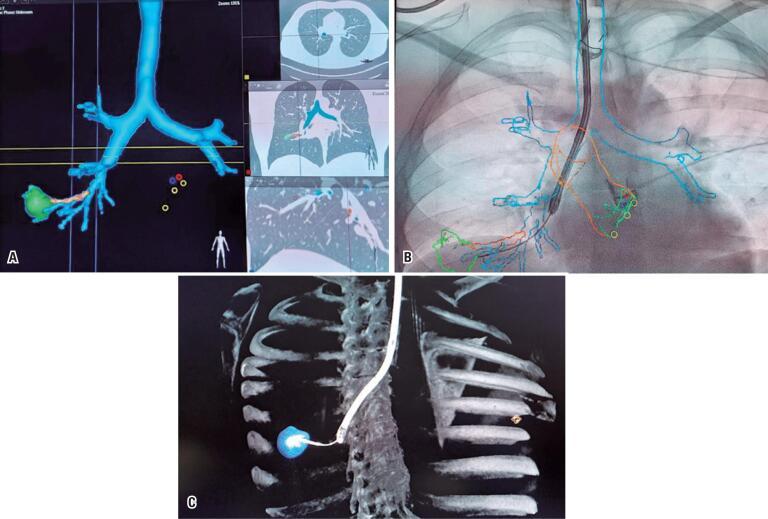
Pre- and intra-procedure imaging evaluation. (A) Pre-computerized tomography procedure based planning: airways on blue, target airway on orange, target lesion on green; (B) Intra-procedure real-time fluoroscopy with 3D roadmap guided bronchoscopy, and endobronchial ultrasound with radial transducer. Ultrasound transducer on the right way guided by 3D road map; (C) 3D software reconstruction after procedure showing the correct positioning of biopsy needle

Dear Editor,

The article “Endobronchial ultrasound: minimally invasive technology to aid in the diagnosis of thoracic diseases”,^(1)^ contributes to technical-diagnostic evolution of pulmonary lesions. Endobronchial ultrasound with a radial transducer enabled to perform cytopathological examination and transbronchial biopsy of small lung lesions. Bronchoscopic radial ultrasound and fluoroscopic images are usually combined procedures. The ability to target small peripheral lesions is technically complex due to the challenging localization of small airways in relation to lung lesions. For this reason, we began to perform the procedure in an interventional radiology suite, with the Philips Azurion angiograph by using image fusion software (Allura Clarity Philips, Netherlands) and cone beam tomography (Figure 1A and B). In this way, we can perform roadmap navigation, and identify bronchial ramifications and the lesion (Figure 2A to C). This technology allowed to reach smaller and more peripheral lesions, reduce radiation for patients and healthcare professionals, and make endobronchial intervention more accessible and less morbid.
